# Supplementary material for: Xylanase Production by Talaromyces amestolkiae Valuing Agroindustrial Byproducts
Source: BioTech (Basel). 2022 May 17;11(2):15. doi: 10.3390/biotech11020015 (PMC9264394; doi:10.3390/biotech11020015)
Supplement: Supplementary file 1 [file biotech-11-00015-s001.zip › biotech-1671813-supplementary.pdf]

Article

Xylanase production by *Talaromyces amestolkiae* valuing agro-industrial byproducts

Giórgia S. Barbieri [1], Heitor B. S. Bento [1], Fernanda de Oliveira [1], Flávio P. Picheli [1], Fernando Masarin [1], Lídia M. Dias [1], Valéria C. Santos-Ebinuma [1,*].

[1] Department of Engineering Bioprocess and Biotechnology, School of Pharmaceutical Sciences, São Paulo State University (UNESP), Rodovia Araraquara-Jau km. 01, CEP:14800-903, Araraquara SP, Brazil.

* Correspondence: valeria.ebinuma@unesp.br; Tel: 55-16-3301-4647

Supplementary material

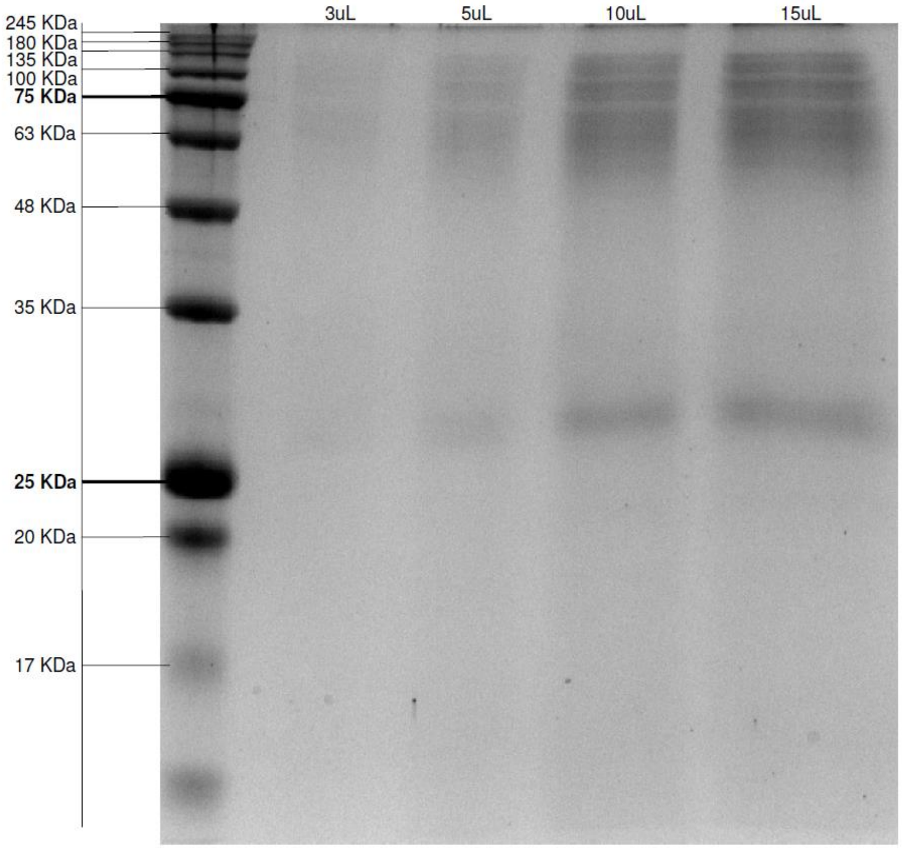

Figure S1. SDS-PAGE electrophoresis of the enzyme produced by T. amestolkiae cultivated in wheat-bran-based medium. The samples were prepared by resuspending the lyophilized extract in distilled water 0.01 % (w/v) which was centrifuged at 2000xg/15 min. The sample was incubated in Laemmli buffer (1:1) at 100 °C, 10 min for protein denaturation. The samples were added to SDS-PAGE 5%/10% (Mini-protean tetra cell BioRad®) under the conditions of 100 V, 20 mA. The molecular weight standard used was the BLUeye Sigma® (11-245 KDa) composed of 12 bands.
